# Potential of an Eco-Sustainable Probiotic-Cleaning Formulation in Reducing Infectivity of Enveloped Viruses

**DOI:** 10.3390/v13112227

**Published:** 2021-11-04

**Authors:** Maria D’Accolti, Irene Soffritti, Francesco Bonfante, Walter Ricciardi, Sante Mazzacane, Elisabetta Caselli

**Affiliations:** 1 Section of Microbiology, Department of Chemical, Pharmaceutical and Agricultural Sciences, LTTA, University of Ferrara, Via Luigi Borsari 46, 44121 Ferrara, Italy; maria.daccolti@unife.it (M.D.); irene.soffritti@unife.it (I.S.); 2 CIAS Research Center, University of Ferrara, Via Saragat 13, 44122 Ferrara, Italy; sante.mazzacane@unife.it; 3 Istituto Zooprofilattico Sperimentale delle Venezie, IZSVe, Viale dell’Università 10, 35020 Legnaro, Italy; fbonfante@izsvenezie.it; 4 Faculty of Medicine and Surgery, Università Cattolica del Sacro Cuore, Largo Francesco Vito 1, 00168 Roma, Italy; walter.ricciardi@unicatt.it

**Keywords:** enveloped virus decontamination, SARS-CoV-2 inactivation, prevention, infection control, eco-friendly disinfection

## Abstract

The COVID-19 pandemic has deeply influenced sanitization procedures, and high-level disinfection has been massively used to prevent SARS-CoV-2 spread, with potential negative impact on the environment and on the threat of antimicrobial resistance (AMR). Aiming to overcome these concerns, yet preserving the effectiveness of sanitization against enveloped viruses, we assessed the antiviral properties of the Probiotic Cleaning Hygiene System (PCHS), an eco-sustainable probiotic-based detergent previously proven to stably abate pathogen contamination and AMR. PCHS (diluted 1:10, 1:50 and 1:100) was tested in comparison to common disinfectants (70% ethanol and 0.5% sodium hypochlorite), in suspension and carrier tests, according with the European UNI EN 14476:2019 and UNI EN 16777:2019 standards. Human alpha- and beta-coronaviruses hCoV-229E and SARS-CoV-2, human herpesvirus type 1, human and animal influenza viruses, and vaccinia virus were included in the study. The results showed that PCHS was able to inactivate 99.99% of all tested viruses within 1–2 h of contact, both in suspension and on surface. Notably, while control disinfectants became inactive within 2 h after application, the PCHS antiviral action persisted up to 24 h post-application, suggesting that its use may effectively allow a continuous prevention of virus spread via contaminated environment, without worsening environmental pollution and AMR concern.

## 1. Introduction

The COVID-19 pandemic, caused by the new SARS-CoV-2 human coronavirus has deeply influenced the habits relative to hygiene and sanitization, shining light on the risk associated with environmental virus contamination, especially in the hospital environment. COVID-19 has in fact spread worldwide, causing at the moment over 244 million confirmed cases and 4.96 million deaths [1]. The SARS-CoV-2 infection is mostly transmitted via respiratory droplets, but the virus has been reported to persist up to days on inanimate hard surfaces, at least in controlled laboratory conditions [2,3], suggesting a potential contribution to transmission of infection through direct contact with surfaces and fomites contaminated by droplets or other body fluids [4,5,6,7,8,9]. Although fomite transmission is difficult to prove definitively [10], a few cases have been reported [11,12] and infection risk has been evaluated linked to possible hand-to-fomite transmission [8,13,14].

Consistently with these data, WHO has proposed preventive measures, and high-level virucidal chemical disinfectants have been mandatorily introduced by regulatory bodies for surface cleaning of indoor environments, including healthcare and non-healthcare settings, as interim recommendations to combat the COVID-19 health emergency [10,15]. However, based on what has been observed on other microorganisms, the disinfectant action may be temporary and not able to prevent recontamination, which occurs continuously due to continuous spread by people present in the confined environment [16]. Despite the initial rapid microbes inactivation, the sanitized surfaces may be rapidly recontaminated, potentially becoming a new transmission source. Furthermore, the excessive use of disinfectants may represent a threat for people [17] and controversies exist about the need of disinfectants instead of cleansers, especially in low-risk healthcare or non-healthcare environments [18,19]. Last, the massive use of disinfectants could negatively impact on urban environments and wildlife [20], as well as aquatic ecosystems [21], and several chemical compounds used for disinfection have been proven to select or induce antimicrobial resistance (AMR) in pathogens, including those known to have an important impact on COVID-19 clinical care [22,23,24,25]. Considering that AMR microbes can complicate the care of COVID-19 patients, and that AMR alone is already killing millions of people each year (over 37,000 people only in the European Union), it is apparent that a further spread of AMR might worsen the toll of future pandemics. Consequently, there is an urgent need for simple, efficient, low-impact, and possibly low-cost procedures to ensure a durable sanitization of treated surfaces, overcoming the side effects linked to chemical disinfection. 

Besides SARS-CoV-2, several enveloped viruses have been similarly shown to retain infectivity for long periods on hard surfaces, depending on virus type, surface characteristics, temperature, and humidity [26], including human coronaviruses, influenza viruses, and herpesviruses [27,28,29]. Parainfluenza viruses, hepatitis B and C viruses, and HIV-1 were also reported to persist on surfaces and fomites [30], which may represent possible virus *reservoirs* and transmission vectors to susceptible humans [28,31]. Consistently with the evidence reporting virus presence and persistence in the hospital environment [30,32,33], showing a theoretical risk of virus transmission to hospitalized patients, the strategies aimed to prevent and control infections include environmental hygienization as part of this process. Notably, until recently the virus component of the hospital microbiome was not considered in the monitoring strategies to counteract the onset of healthcare-associated infections (HAI) in clinical settings, as it is done for bacterial and fungal ones, although a viral infectious risk exists even if minimal.

Among the viruses that might be transmitted by the contaminated environment, human coronaviruses (enveloped single stranded, positive sense RNA viruses), which can cause mild to severe respiratory infections [34], can persist on different inanimate surface types, remaining infectious from 2 h up to 9 days at room temperature [3,4]. Similarly, the influenza viruses (enveloped single stranded, negative sense RNA viruses), whose type A is the most virulent among the four influenza types [35], are able to retain infectivity up to 48 h on smooth surfaces [36,37], and the H5N1 strain can persist beyond 13 days on glass and steel at low relative humidity and temperature [38]. Furthermore, the human herpesvirus Herpes simplex virus-1 (HSV-1; an enveloped double strand DNA virus), the causative agent of oral and genital herpes, can survive on dry inanimate surfaces from few hours to one week [28], and its transmission can cause a wide range of infections, from mild to life-threatening ones in immune-immature individuals or immune-compromised patients [39].

Based on the ability of many viruses to maintain infectivity on inanimate surfaces, the control of environmental viral contamination represents a key point to address. Current guidelines for COVID-19 management suggest using high level disinfectants, mostly 0.1–0.5% sodium hypochlorite (NaClO) and 1% hydrogen peroxide (H_2_O_2_), for sanitization [40]. However, despite their rapid action [41], there is no warranty of a long-lasting action to stably maintain the environment decontaminated. Rather, based on previous reports by us and others [16,42,43,44], the sanitized environment could be rapidly recontaminated, leading to a persistent level of contamination for most of the day. 

Aiming to obtain a long-term effective cleaning procedure, stably reducing viral contamination without impacting on environmental pollution and AMR, we tested the antiviral properties of an eco-sustainable probiotic-based sanitation system (PCHS, Probiotic Cleaning Hygiene System), that was previously shown to prevent pathogen recontamination by stably remodulating the hospital microbiome. Its action, based on the biological properties of selected probiotic *Bacillus* species contained in an eco-friendly detergent, stably reduced resistant pathogens (−80%) [16,22,45,46,47,48] and associated infections (−52%) [44,46], and also had a relevant positive impact on antimicrobial consumptions (−60%) and therapy costs (−75%) [46]. Based on these observations, here we assessed the PCHS antiviral efficiency on different enveloped viruses known to be able to persist long on surfaces, namely human coronaviruses HCoV-229E and SARS-CoV-2, HSV-1, type A influenza viruses of human (human H3N2) and animal (avian H10N1 and swine H1N2) origin, and the modified Vaccinia virus Ankara (MVA), this virus being the most resistant among enveloped viruses and for this reason mandatorily included in the European standard procedures used to assess the antiviral properties of disinfectants. The assays were carried out in vitro, following the European standard norms for suspension and surface tests [49], testing both the decontaminating and preventing activity of PCHS in comparison with standard disinfectants.

## 2. Materials and Methods

### 2.1. Probiotic-Based Detergent

The Probiotic Cleaning Hygiene System (PCHS)(Copma Scrl, Ferrara, Italy) used in all the assays was previously described [22]. Briefly, it consists of a patented EU Ecolabel (https://eur-lex.europa.eu/legal-content/EN/TXT/?uri=CELEX:32010R0066, accessed on 23 October 2021) detergent containing 10^7^ CFU/mL spores of selected probiotics belonging to the *Bacillus* genus (namely *B. subtilis*, *B. pumilus*, and *B. megaterium* species). PCHS was tested at 1:10, 1:50, and 1:100 dilution in sterile distilled water.

### 2.2. Viruses and Cells

The enveloped viruses and appropriate target cells used for virus inocula preparation, virus titration, and standard inactivation assays, are summarized in Table 1.

MRC-5 cells were cultured in Eagle Minimal Essential Medium Eagle (EMEM)(Gibco, Grand Island, NY), whereas the other cell lines were grown in Dulbecco Minimal Essential Medium (DMEM)(Gibco, Grand Island, NY). All the cell lines were expanded at 37 °C + 5% CO_2_ in the appropriate culture medium supplemented with 10% foetal bovine serum (FBS), 2 mM L-Glutamine, 100 U/mL penicillin, and 100 µg/mL streptomycin (cell culture complete medium) (Gibco, Grand Island, NY).

Virus stocks were obtained by infecting specific 90% confluent target cells, subsequently incubated at the appropriate temperature (35 °C for hCoV-229E and 37 °C for all the other viruses) + 5% CO_2_ in culture medium additioned with 2% FBS. Infected cell cultures were incubated for different times until appearance of cytopathic effect (CPE) involving >80% of cultured cells, that corresponded to: 2 days for MVA and HSV-1, 5 days for SARS-CoV-2, 3 days for influenza viruses, and 7 days for hCoV-229E. At the end of the incubation time, cells and culture supernatants were collected. Cells were lysed by 3 cycles of rapid freezing/thawing in liquid nitrogen and 37 °C, interspersed with 30 s pulse-vortex. Cell lysate was then added to culture supernatant and viral particles were recovered by centrifugation at 20,000× *g* for 45 min at 4 °C. The virus pellets were suspended in 1 mL of PBS + 1% bovine serum albumin (BSA), then frozen and maintained at −80 °C until use. The virus stock titre was determined by infecting the appropriate target cells seeded in 96-well plates, using the same culture conditions used for virus stock preparation, and evaluating the 50% tissue culture infectious dose (TCID_50_) per ml by the standard Spearman-Karber method, as previously described [50,51]. Briefly, serial dilution of the viral inocula were added to sestuplicate samples of target cells seeded in 96-well plates, and CPE was recorded after the adequate incubation time. Virus titre was calculated by the following formula, to directly estimate the 50% end point.

(1) $$\mathrm{LogID}50=\mathrm{Log}\;(\mathrm{highest}\;\mathrm{dilution}\;\mathrm{giving}\;100\%\;\mathrm{CPE})+0.5\;-\frac{\mathrm{total}\;N{}^{\circ}\;\mathrm{test}\;\mathrm{units}\;\mathrm{showing}\;\mathrm{CPE}}{N{}^{\circ}\;\mathrm{test}\;\mathrm{units}\;\mathrm{per}\;\mathrm{dilution}}$$

All virus stocks contained around 10^8^ TCID_50_/_mL_, as calculated by the Spearman-Karber method.

### 2.3. Antiviral Activity: Suspension Tests

The antiviral activity of PCHS in suspension was assayed following the European standard procedure UNI EN 14476:2019, as indicated by the Technical Committee 216 (TC216) “Chemical disinfectants and antiseptics” of the European Committee for Standardization (CEN), which has been developing methods for testing the efficacy of disinfectants in Europe since 1989 [49,52]. Briefly, 10 µL of virus stock suspension (corresponding to 10^5^–10^7^ TCID_50_, depending on virus type) were added to 90 µL of the appropriate dilution of PCHS (1:10, 1:50 and 1:100), in the presence of 0.3% of BSA, to mimic the conditions that could be found on a hospital surface (“clean” conditions). Negative and positive controls were respectively represented by culture medium and 70% ethanol (EtOH). The suspension was incubated at room temperature for 1, 2, 4, 8, and 24 h; then it was collected, immediately diluted in 0.9 mL cold medium + 2% FBS (neutralization step), filtrated (0.45 µm) to remove the probiotic component, and then serially diluted (10-fold dilution) in cold medium + 2% FBS for titration of the residual virus amount by the Spearman-Karber method. Infected cells were then incubated at the appropriate temperature in the presence of 5% CO_2_ for the time needed to evidence virus CPE. Representative CPE pictures are shown in Supplementary Materials Figure S1. Infectious titre was expressed as TCID_50_/_mL_. Each experimental condition was assessed in duplicate and the collected results represent the mean values of three independent assays. All the experiments with SARS-CoV-2 were performed in a BSL-III laboratory. Experimental controls and cytotoxicity evaluation of PCHS and standard disinfectants were performed in all cell types used in the assays, following the protocol indicated in the standard procedures.

### 2.4. Antiviral Activity: Surface Tests

The antiviral ability of PCHS on hard non-porous surfaces was assessed following the European standard procedure UNI EN 16777:2019 [53] and using MVA and hCoV229E as the target viruses. The rule provides for evaluations to be performed on stainless steel sterile discs of 2 cm diameter, which were contaminated in three different conditions, to assess respectively: (1) the PCHS ability to decontaminate previously virus-contaminated surfaces, (2) the short-term ability of PCHS-treated surfaces to inactivate a subsequent virus contamination, (3) the long-term action of PCHS in stably preventing a subsequent virus contamination. Each assay was performed in the presence of 0.3% BSA as the organic load (“clean” conditions). Briefly, in decontamination assays, 100 µL of virus inoculum (corresponding to 10^6^ TCID_50_) were seeded on carrier surface with a micropipette, and the drop was spread on a surface area of about 1 cm diameter and left to dry at room temperature, then immediately covered with 100 µL of PCHS previously diluted 1:10, 1:50, and 1:100 in water. EtOH 70% and culture medium were used as positive and negative controls, respectively. After 1, 2, 4, 8, and 24h, 0.9 mL (10-fold dilution) of ice cold medium + 2% FCS were added to collect the residual virus. The collected medium was then filtered (0.45µm) to remove probiotic component and serially diluted (10-fold) in cold medium + 2% FCS. Each dilution was transferred to 96-well microplates (0.1 mL/well) containing a 90% confluent monolayer cells, which were then incubated at the appropriate conditions to evaluate the residual virus titre by the Spearman-Karber method. In the prevention assay, 100 µL of 1:10, 1:50, and 1:100 diluted PCHS were seeded on surface and left to dry at room temperature. EtOH 70% and culture medium were used as positive and negative controls, respectively. Immediately after drying, the surfaces were contaminated with 100 µL of virus inoculum (10^6^ TCID_50_). After 1, 2, 4, 8, and 24h, samples were collected and titrated as described for the decontamination assay. The long-term ability of PCHS in preventing virus contamination was assayed by seeding 100 µL of 1:10, 1:50, and 1:100 diluted PCHS on surface and then contaminating the treated surface after 1, 2, 4, 8, and 24h with 100 µL of virus inoculum (10^6^ TCID_50_). EtOH 70% and sodium hypochlorite (NaClO) 0.5% were used as positive controls; culture medium was used as a negative control. Virus inoculum was left in place for 2 h and then collected and titrated as described for the previous assays. Experimental controls and cytotoxicity evaluation of PCHS and standard disinfectants were performed on all cell types used in the assays, following that indicated in the standard procedures. Both EtOH and NaClO were cytotoxic at 10^−1^ dilution on all used cell lines, whereas PCHS did not show cell toxicity; direct comparison of virus titre was thus performed on the dilutions from 10^−2^ onwards.

### 2.5. Analysis of Probiotic Enzymatic Activity

The *Bacillus* strains contained in the PCHS detergent (namely *B. subtilis*, *B. pumilus* and *B. megaterium*), previously culturally isolated, were assessed by the API-ZYM system (BioMérieux, Florence, Italy) for their ability to produce enzymes potentially useful to degrade virus components, following the manufacturer’s instructions.

### 2.6. Statistical Analysis

Statistical analyses were performed with Agilent GeneSpring GX v11.5 software (Agilent Technologies, Santa Clara, CA, USA) and R (R 2019, R Core Team, available as free software at https://www.r.project.org/, accessed on 10 May 2021) by Student’s *t*-test. A *p*-value ≤ 0.05 was considered significant.

## 3. Results

### 3.1. PCHS Antiviral Activity in Suspension

**Figure 1:**
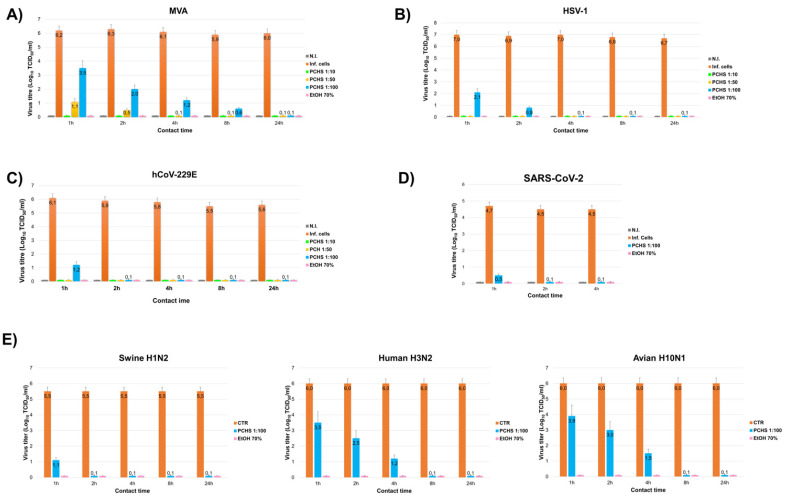
PCHS antiviral activity in suspension tests performed on the indicated enveloped viruses. (**A**) modified Vaccinia virus (MVA) virus titre after contact with PCHS at the indicated dilutions, as measured in BHK target cells. (**B**) HSV-1 virus titre after contact with PCHS at the indicated dilutions, as measured in target Vero cells. (**C**) hCoV-229E virus titre after contact with PCHS at the indicated dilutions, as measured in MRC-5 target cells. (**D**) SARS-CoV-2 virus titre after contact with PCHS (dilution 1:100), as measured in Vero target cells. (**E**) human and animal influenza virus titres after contact with 1:100-diluted PCHS, as measured in swine H1N2, human H3N2 and avian H10N1 strains were used, and residual virus titre was measured in MDCK target cells. Positive control of virus inactivation was represented by 70% EtOH in each assay. Results are expressed as Log_10_ TCID_50_/_mL_, and represent the mean ± SD values of duplicate samples from three independent assays for each virus type.

The results obtained by measuring the antiviral activity of PCHS on the indicated enveloped viruses, in suspension conditions (according to the standard European procedure UNI EN 14476:2019), showed that PCHS could efficiently inactivate all the tested viruses, regardless of the type of virus used (Figure 1), including MVA, which is the only enveloped virus included mandatorily in the standard guidelines, considered the most resistant among the viruses provided with envelope. In detail (Figure 1A), the higher concentrations of PCHS gave a >4 Log inactivation of MVA within 1 h of contact (−6.1 and −5.1 Logs for 1:10 and 1:50 dilutions, respectively), whereas the 1:100 dilution provided a −2.7 Log in 1 h but a >4 Log decrease within 2 h (−4.3 Logs), meeting the standard UNI EN 14476:2019 rules to fulfil the efficacy requirements for a product with antiviral activity against a specific virus type. The reduction of infectious virus titre increased in a time-dependent manner at subsequent incubation times (4, 8, and 24 h).

Figure 1B shows the results obtained using HSV-1, which confirmed and extended those observed with MVA. In fact, a >4 Log virus inactivation was observed with any PCHS dilution within 1 h of contact. The high-diluted product (1:100) provided a 4.9 Log decrease within 1 h, and higher PCHS concentrations (1:10 and 1:50 dilutions) completely inactivated the original virus titre, recording a 7.0 Log reduction. Similarly, the results obtained against the human alpha-coronavirus hCoV-229E (Figure 1C) showed a 4.9 Log inactivation in 1 h of contact with the dilution 1:100, and no residual virus with the higher concentrations (−6 Logs). At later times (2, 4, 8, and 24 h), hCoV-229E inactivation was complete, the residual virus being undetectable.

Consistently, PCHS exhibited a similar antiviral activity also against SARS-CoV-2 (Figure 1D), which was inactivated >4 Log (−4.1 Logs) by the dilution 1:100 within 1 h of contact. The inactivation was complete at 2 and 4 h post contact, as no residual virus was detectable at those times. Based on the obtained results, and similar to that performed in SARS-CoV-2 assays, only the 1:100 PCHS dilution was tested against influenza viruses, using contact times of 1, 2, 4, 8, and 24 h. The results (Figure 1E) showed that human and animal strains were differently sensitive to PCHS activity, with swine H1N2 strain being the most susceptible and the avian H10N1 strain most resistant. The swine H1N2 virus was in fact inactivated >4 Log by PCHS within 1 h (−4.4 Logs), whereas the human H3N2 strain showed a decrease of 2.5, 3.5, and 4.9 Logs, and the avian H10N1 strain a decrease of 2.1, 3, and 4.5 Logs after 1, 2, and 4 h, respectively.

### 3.2. PCHS Antiviral Activity on Surface

Since in the suspension method the viruses come into contact with a large amount of the disinfectant, which may render them easier to inactivate, the antiviral activity of PCHS was also assessed in carrier tests, performed according to the UNI EN 16777:2019 standard European procedure. Based on the results obtained in suspension tests, only hCoV-229E and MVA viruses were used in the assays, to include a human coronavirus similar to SARS-CoV-2 and a highly resistant enveloped virus. Three different conditions were tested, to assess respectively: (1) the decontaminating ability of PCHS, (2) the PCHS ability to prevent virus contamination by PCHS, (3) the long-term stability of PCHS activity in preventing virus contamination.

**Figure 2:**
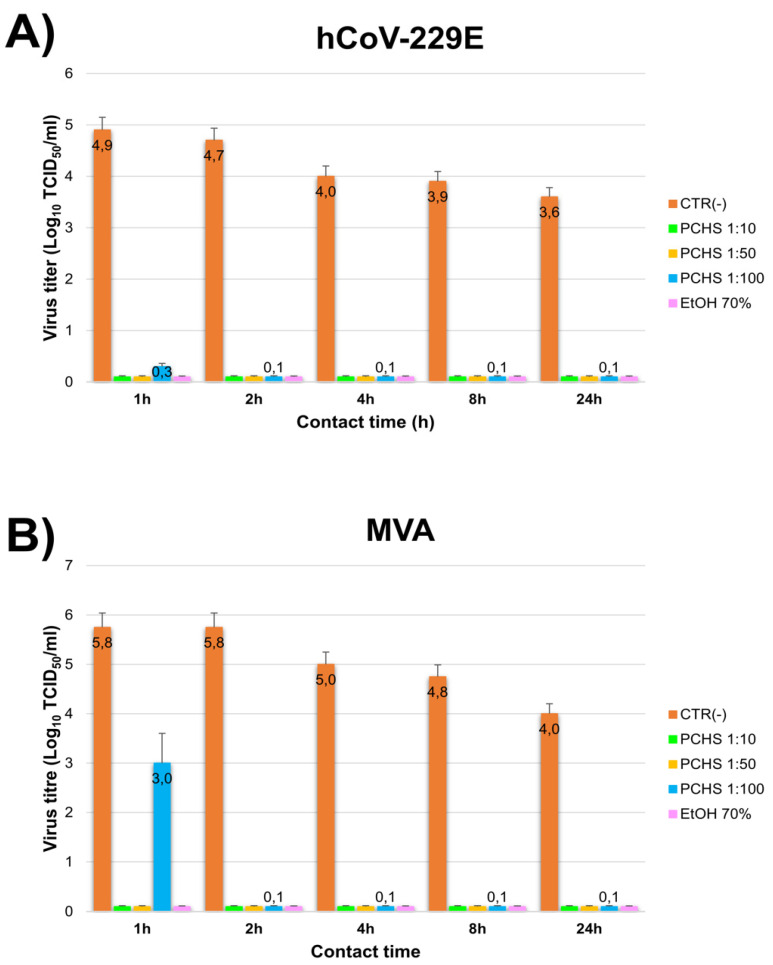
PCHS activity in surface decontamination tests. Assays were performed by applying the indicated PCHS dilutions (1:10, 1:50, and 1:100) to surfaces previously contaminated with the indicated viruses. Samples were collected 1, 2, 4, 8, and 24 h, and the residual virus titre was evaluated by Spearman Karber method. 70% EtOH was used as control; results are expressed as Log_10_ TCID_50_/_mL_, and represent the mean ± SD values of duplicate samples from three independent assays. (**A**) hCoV-229E results. (**B**) MVA results.

The results of decontamination-type assays showed that, similar to that observed in suspension tests, any dilution of PCHS could completely inactivate hCoV-229E (>4 Log decrease) within 1 h, whereas MVA was totally inactivated in 1 h by 1:10 and 1:50 dilutions and in 2 h by 1:100 diluted PCHS (Figure 2). The lowest PCHS concentration induced however a 2.5 Log decrease of MVA titre within 1 h.

**Figure 3:**
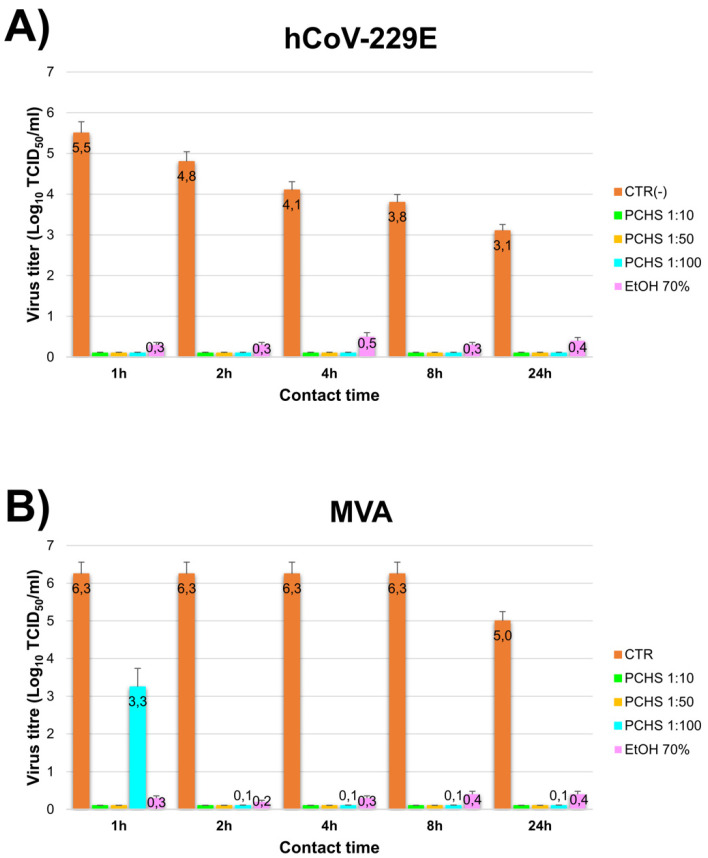
PCHS ability to prevent virus contamination on treated surface. Assays were performed by applying PCHS on surface (dilutions 1:10, 1:50, and 1:100), left to dry, and immediately contaminating treated surfaces with the indicated viruses. After 1, 2, 4, 8, and 24 h, samples were collected and the residual virus titre was evaluated by Spearman-Karber method. As control, 70% EtOH was used; results are expressed as Log_10_ TCID_50_/_mL_, and represent the mean ± SD values of duplicate samples from three independent assays. (**A**) hCoV-229E results. (**B**) MVA results.

Next, the ability of PCHS-treated surfaces to prevent a subsequent virus contamination was assessed. To this purpose, PCHS was first applied on surface and left to dry, then the treated surface was contaminated with the virus inoculum, whose residual titre was evaluated after 1, 2, 4, 8, and 24 h of contact. The results, summarized in Figure 3, showed that PCHS-treated surfaces could inactivate subsequently contaminating viruses, providing complete inactivation of hCoV-229E within 1 h at any PCHS dilution, whereas MVA was completed inactivated in 1 h by 1:10 and 1:50 diluted PCHS and in 2 h by 1:100 diluted PCHS, respectively. After 1 h of contact, however, 1:100 diluted PCHS gave a 3-Log decrease of MVA titre. Of note, 70% EtOH appeared less active than PCHS in inactivating both viruses, suggesting that evaporation caused by drying on surfaces induced a partial loss of action.

**Figure 4:**
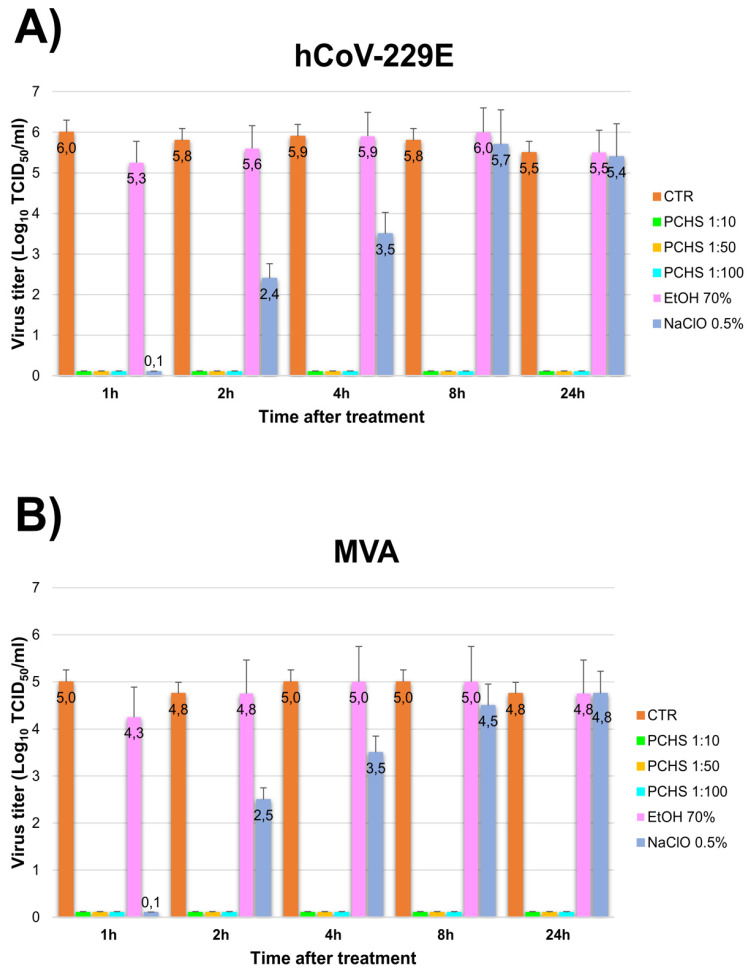
Long-term ability of PCHS to prevent virus contamination on treated surface. Assays were performed by applying PCHS on surface (dilutions 1:10, 1:50, and 1:100), left to dry and left on surface for 1, 2, 4, 8, and 24 h. Afterwards, treated surfaces were contaminated with the indicated viruses. After 2 h, samples were collected and the residual virus titre was evaluated by Spearman-Karber method. As controls, 70% EtOH and 0.5% NaClO were used; results are expressed as Log_10_ TCID_50_/_mL_, and represent the mean ± SD values of duplicate samples from three independent assays. (**A**) hCoV-229E results. (**B**) MVA results.

Lastly, the long-term ability of PCHS to prevent virus contamination was assessed on surfaces. PCHS (diluted 1:10, 1:50, and 1:100) was applied on surfaces and left to dry, then virus inocula were added to treated surfaces after 1, 2, 4, 8, and 24 h. Viruses were left in place for 2 h and then collected to measure residual virus titre (Figure 4).

Notably, PCHS-treated surfaces completely inactivated both viruses even 24 h after treatment, whereas EtOH- and NaClO-treated surfaces did not maintain their inactivating ability over time. EtOH lost its inactivating activity within 1 h and NaClO gradually lost its virucidal activity from 2 h on both MVA (−2.25 Logs at 2 h) and hCoV-229E (−3.4 Logs): at that time, in fact, the NaClO activity resulted below the threshold needed to define a compound as virucidal (−4 Logs). At longer times the antiviral activity of both chemical disinfectants disappeared completely.

### 3.3. Enzymatic Activity of PCHS-Probiotics

To elucidate the possible contribution of probiotics in virus inactivation, PCHS-derived *Bacillus* strains were analyzed for their enzymatic activity, in order to highlight any eventual production of enzymes capable of degrading viral components. The three *Bacillus* strains included in the PCHS detergent (*B. subtilis, B. pumilus,* and *B. megaterium*) were individually tested by the API-ZYM system, allowing to identify and quantify simultaneously 19 different enzymatic activities. The results, summarized in Table 2, showed that each strain exhibited several enzymatic activities, including alkaline and acid phosphatase, esterase and esterase lipase, leucine and valine arylamidase (*B. pumilus* and *B. megaterium*), α-chimotrypsin (*B. pumilus* and *B. megaterium*), naphtol-phosphohydrolase, α- and β-galactosidase (absent respectively in *B. megaterium* and *B. subtilis*), α- and β-glucosidase, N-acetyl-β-glucosamidase, and α-mannosidase (*B. pumilus* only). The presence of enzymes capable of processing lipids, proteins, and sugars, support the hypothesis that such probiotics could chemically degrade the outer components of enveloped viruses, achieving virus inactivation.

## 4. Discussion

The COVID-19 pandemic, caused by SARS-CoV-2, has profoundly influenced sanitization procedures and the massive use of disinfectants has been indicated to prevent virus spread. On the other hand, contamination of viral origin has been reportedly evidenced for several viruses beside SARS-CoV-2 [33], possibly contributing to healthcare-associated infections (HAIs) of viral origin [32], including both enveloped and non-enveloped viruses associated with respiratory, muco-cutaneous, blood-borne, and pediatric diseases [32,54]. However, the use of chemical disinfectants has tremendously increased during the COVID-19 pandemic, to fight SARS-CoV-2 spread [3,40,41,55,56], and also new disinfection systems have been proposed [57]. Regardless, some effective chemical disinfectants can be toxic to humans and must be applied only in the absence of people [58], and even with optimal cleaning and disinfecting practices, recontamination of the environment and equipment occurs quickly, due to the temporary action of disinfectants, finally allowing recontamination [16,59,60]. Furthermore, as pointed out by the WHO in a recent editorial [61], the current massive use of chemical disinfectants may exacerbate both environmental pollution and AMR [24,62,63].

In contrast, we recently reported that a sanitation approach based on the microbiome-balance principle (PCHS, Probiotic Cleaning Hygiene System), could stably and efficiently counteract pathogens compared to chemical disinfectants, through competitive exclusion mechanisms [64], decreasing pathogens 80% more than chemical disinfectants [16,22,47,65], without selecting resistant microbes but rather decreasing the existing AMR up to 99.9% [46,48,66], and also decreasing the HAI incidence (−52%) and the HAI-associated drug consumption (−65%) [46].

Based on these premises, and in consideration of the current emergency linked to COVID-19, here we aimed to assess the antiviral potential of PCHS against the enveloped viruses potentially contaminating the healthcare setting, including SARS-CoV-2. PCHS was tested following suspension and surface procedures, according with the European standard UNI EN 14476:2019 and UNI EN 16777:2019. Indeed, both procedures prescribe the use of non-enveloped viruses and only MVA among the enveloped ones (since it is considered the most resistant enveloped virus); however, since our study aimed to analyze the enveloped viruses potentially able to persist on surfaces and similar to SARS-CoV-2, we included, beside MVA, the human coronaviruses hCoV-229E and SARS-CoV-2, the human herpesvirus HSV-1 and three human/animal type A influenza viruses. Three different concentrations of PCHS were tested, including a lower one that is used for routine cleaning (1:100 dilution) and two higher concentrations (1:10 and 1:50 dilutions), comparing their activity with that of 70% EtOH and 0.5% NaClO, two common disinfectants indicated for decontamination during the pandemic. Based on standard procedures evaluating the virucidal properties of a product, we considered a ≥4-Log_10_ decrease of virus titre obtained within 1 h as the threshold to establish the antiviral activity of the product against a specific virus.

The results showed that PCHS was active on all enveloped viruses at all dilutions in a time-dependent manner. In particular, the 1:100 dilution completely inactivated within 1 h all tested viruses except for MVA, which was inactivated in 2 h. A variable situation was observed with influenza viruses, confirming that previously reported for chemical disinfectants, showing different activity depending on virus type and host origin [67]. In fact, the 1:100-diluted PCHS inactivated swine virus within 1 h, whereas inactivation of human and avian viruses required 2–4 h, suggesting that higher PCHS concentrations should be used against those strains to obtain inactivation in shorter times.

The carrier/surface test results, performed on hCoV-229E and MVA, evidenced a high inactivation activity both in decontamination and prevention conditions. Interestingly, while 70% EtOH and 0.5% NaClO became inactive within 1 and 2 h, respectively, PCHS-treated surfaces maintained their antiviral properties even 24 h after PCHS application, suggesting that a daily PCHS sanitation (as usually performed in routine cleaning) may assure a continuous stably decontaminated environment.

The surfactants contained in detergent are known to disrupt and damage the envelope of viruses [55,68], and may thus account at least in part for the PCHS antiviral action. However, detergent’s long-lasting action on surfaces is difficultly attributable to surfactants only, and we hypothesized that the production of enzymes by the PCHS-*Bacillus* strains spread on the treated surface may be responsible for the continuous action. Accordingly, the analysis of the enzymes produced by the three PCHS-derived *Bacillus* species (*B. subtilis*, *B. pumilus* and *B. megaterium*) revealed several enzymatic activities potentially able to degrade the virus shell components, including lipids, protein, and sugar residues. Consistent with this, the *Bacillus* genus is reportedly known as one of the most important bacterial sources of enzymes with remarkable properties, such as high resistance to extreme temperatures, pH, organic solvents, and oxidizing agents. *Bacillus* enzymes have been long used in medicine, biofilm destruction, animal feed, agriculture, degradation of feathers, wool, and hair [69,70], thus supporting their ability to also degrade the outer virus components. Indeed, such activities have been previously associated with an effective 99.9% reduction of microbial contamination [71], suggesting that they may be relevant also against viruses. In addition, the bacteria belonging to *Bacillus* genus possess sporulation capacity that renders them resistant to harsh environmental conditions and usable in concentrated detergents, which makes them interesting for medical and industrial purposes. Lastly, PCHS is a low-cost affordable system, which is also important from a cost-saving perspective [72], and renders it potentially applicable in several non-sanitary environments, including schools, offices, public transportation, as well as in low-income countries. Of note, the UNI EN 14885:2018 indicates two types of disinfection in human medicine: a fast one (within 5 min) for critical patient area, and a slow one (within 1 h) for the other areas [73]. To this regard, PCHS would provide a slow disinfection, but its stable 24 h decontaminating activity suggests that it may long protect the treated environment, meeting also the recent indications of CDC regarding the possibility, in the absence of people with confirmed/suspected COVID-19, that a daily cleaning can be sufficient to remove the virus from surfaces and maintain a healthy facility [19]. For non-healthcare environments it would however be relevant to test PCHS effectiveness also in the presence of high organic soil (“dirty” conditions), as non-sanitary spaces may have higher amounts of organic matter compared to the hospital space. Moreover, since dry biofilm is an increasing problem on surfaces, it would be of interest to test the action of PCHS on this microbial body. Finally, it will be interesting to expand the analysis also to non-enveloped viruses, to assess the eventual antiviral action against those more resistant viruses.

## 5. Conclusions

In light of its effective long-lasting antiviral activity and characteristics of sustainability, PCHS sanitation may be considered as a novel and safe perspective for the control and prevention of the spread of various enveloped viruses including SARS-CoV-2, also in light of the lack of negative side-effects on the environment health and on AMR concerns.

## Figures and Tables

**Table 1:** Virus trains and target cells used in the assays.

Virus	Strain	Target Cells
modified Vaccinia virus Ankara (MVA)	ATCC VR-1508	baby hamster kidney fibroblast BHK-21 cell line (ATCC CCL-10)
herpes simplex virus type 1 (HSV-1)	ATCC VR-260	monkey kidney fibroblast Vero-E6 cell line (ATCC CRL-1586)
human alpha-coronavirus 229E (hCoV-229E)	ATCC VR-740	human lung fibroblast MRC-5 cells (ATCC CCL-171)
human beta-coronavirus SARS-CoV-2 ^1^	//	monkey kidney fibroblast Vero-E6 cell line (ATCC CRL-1586)
human H3N2 influenza virus ^2^	A/Wisconsin/67/2005	Madin-Darby Canine Kidney MDCK cell line (ATCC CCL-34)
avian H10N1 influenza virus ^2^	A/mallard/Italy/4518/2012	Madin-Darby Canine Kidney MDCK cell line (ATCC CCL-34)
swine H1N2 influenza virus ^2^	A/swine/Italy/4159/2006	Madin-Darby Canine Kidney MDCK cell line (ATCC CCL-34)

^1^ provided by the Institute of Virology and Immunology of the University of Bern, Switzerland ^2^ provided by Istituto Zooprofilattico Sperimentale delle Venezie, IZSVe, Padova, Italy.

**Table 2:** Enzymatic activity of PCHS-*Bacillus* strains (*).

Enzyme	*B. subtilis*	*B. pumilus*	*B. megaterium*
NTC Alkaline phosphatase Esterase	0	0	0
	2	5	4
	4	4	2
Esterase lipase Lipase	4	4	2
	0	0	0
Leucine arylamidase Valine arylamidase Cysteine arylamidase Trypsin	0	3	3
	0	1	1
	0	0	0
	0	0	0
α-chimotrypsin Acid phosphatase	0	1	1
	2	4	5
Naphtol-phosphohydrolase	1	1	2
α-galactosidase	2	1	0
β-galactosidase	0	4	2
β-glucuronidase	0	0	0
α-glucosidase	4	1	2
β-glucosidase	5	5	1
N-acetyl-β-glucosamidase	1	1	1
α-mannosidase	0	3	0
α-fucosidase	0	0	0

(*) The activity of each enzymatic is expressed with a score from 0 to 5 according with the intensity of the reaction, compared to control, as indicated by manufacturer’s instructions.

## Data Availability

All the data presented in this study are contained within the article or its Supplementary Materials.

## Supplementary Materials

The following is available online at https://www.mdpi.com/article/10.3390/v13112227/s1, Figure S1: Representative pictures of the control and infected cells inoculated with the residual virus after 1 and 24 h of contact with PCHS at the indicated dilutions.
