# Supplementary material for: Potential of an Eco-Sustainable Probiotic-Cleaning Formulation in Reducing Infectivity of Enveloped Viruses
Source: Viruses. 2021 Nov 4;13(11):2227. doi: 10.3390/v13112227 (PMC8617880; doi:10.3390/v13112227)
Supplement: Supplementary file 1 [file viruses-13-02227-s001.zip › viruses-1420093-supplementary.pdf]

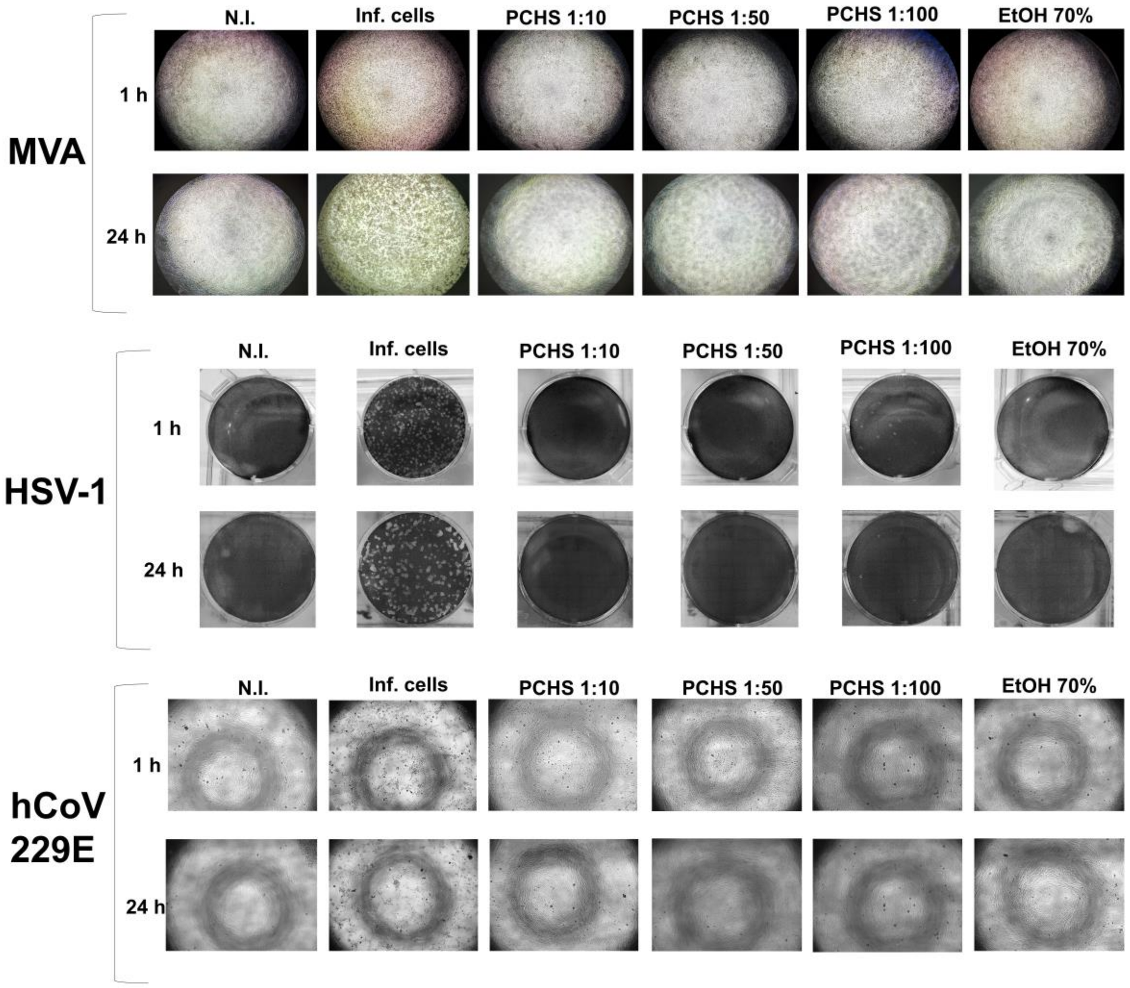

Figure S1: Representative pictures of the control and infected cells inoculated with the residual virus after 1 and 24 hours of contact with PCHS at the indicated dilutions.
